# Frequency of Obstructive Sleep Apnea in Patients With Cystic Fibrosis and Non-cystic Fibrosis Bronchiectasis and Its Association With Clinical Findings

**DOI:** 10.7759/cureus.51224

**Published:** 2023-12-28

**Authors:** Duygu Vezir, Sehnaz Olgun Yıldızeli, Derya Kocakaya, Berrin Ceyhan, Baran Balcan

**Affiliations:** 1 Pulmonary and Critical Care Medicine, Marmara University School of Medicine, Istanbul, TUR; 2 Pulmonary Medicine, Marmara University School of Medicine, Istanbul, TUR; 3 Pulmonary and Critical Care Medicine, Koc University, Istanbul, TUR

**Keywords:** clinical characteristics, disease duration, non-cf bronchiectasis, cystic fibrosis (cf), obstructive sleep apnoea

## Abstract

Objective: This study was designed to assess obstructive sleep apnea (OSA) in adult patients with cystic fibrosis (CF) and non-CF bronchiectasis (BE) and to relate it with clinical characteristics.

Methods: Thirty-five CF (27 years) and 35 non-CF (24 years) BE patients were included. Demographic characteristics, medications, comorbidities, BMI, dyspnea scales, pulmonary functions, sputum cultures, exacerbations, and hospitalizations were recorded. The Epworth Sleepiness Scale (ESS) questionnaire was filled and polysomnography was performed for each patient.

Results: ESS scores did not show any significant difference between CF and non-CF BE patients. Thirty-seven (53%) of all patients had OSA. There was no significant difference in OSA risk between CF and non-CF BE patients (54% vs 51%, respectively). Male gender was found to be a risk factor for OSA (68% of males vs 41% of females, respectively, p:0.026). Total sleep time, sleep efficiency, sleep latency, time spent awake after falling asleep, oxygen desaturation index, apnea-hypopnea-index (AHI), AHI in the supine position, and rapid eye movement phase did not show any significant difference between CF and non-CF patients. CF patients had significantly lower mean oxygen saturation (p:0.001) and lowest oxygen saturation (p:0.0024) levels and higher heart rate (p:0.02) compared to non-CF BE patients. Multiple logistic regression analysis of all patients revealed male gender and disease duration as risk factors for OSA (p:0.023 and p:0.041 respectively).

Conclusion: It is remarkable that more than half of the patients in both CF and non-CF bronchiectasis groups had OSA. Male gender and disease duration were found as risk factors for OSA.

## Introduction

Bronchiectasis (BE) involves the focal or diffuse dilation of bronchi resulting from irreversible damage [1]. While it defines both a clinical disease and a radiological appearance, its development can arise from multiple causes [2]. Etiological factors of BE include primary ciliary dyskinesia, primary immunodeficiencies, systemic diseases (like inflammatory bowel disease and rheumatoid arthritis), foreign body aspiration, childhood lower respiratory tract infections, and notably, cystic fibrosis (CF) [2]. Its typical presentation is a chronic cough accompanied by purulent sputum, often triggered by recurrent lower respiratory tract infections [3].

CF stands out as the predominant autosomal recessive genetic disorder among Caucasians [4]. It is a multisystem ailment rooted in a mutation affecting the cystic fibrosis transmembrane regulator (CFTR) protein, which is integral to the chloride channel and is located on the apical membrane of various epithelia - respiratory, digestive, reproductive, and sweat glands [5].

On another front, obstructive sleep apnea (OSA) is widely recognized by its hallmark repeated breathing cessation during sleep, largely due to full or partial pharyngeal obstruction [6]. Males exhibit a higher prevalence of OSA at 20%, compared to females at 15% [7]. While OSA's cardinal symptoms include witnessed apnea, excessive daytime sleepiness, and snoring, its manifestations can vary based on factors like gender, age, and underlying comorbidities [8].

There is a theory proposing that BE patients might suffer from degraded sleep quality leading to OSA, as a result of recurrent lower respiratory tract infections and sputum accumulation. Pediatric studies have even suggested that OSA might manifest as an initial symptom of CF, especially in milder cases [9]. Furthermore, CF-related conditions like chronic sinusitis and nasal polyps, which increase upper airway resistance, can also contribute to sleep disorders [9-11].

While plenty of literature discusses the link between CF and sleep-disordered breathing (SDB) in children, very few studies have explored the connection between OSA and CF in adults. In this research, our objective is to gauge the prevalence of OSA among adult CF patients and to delineate the relationship between OSA and various parameters like demographics, microbiology, spirometry, and clinical outcomes within this demographic.

## Materials and methods

Study design and participants

**Figure 1:**
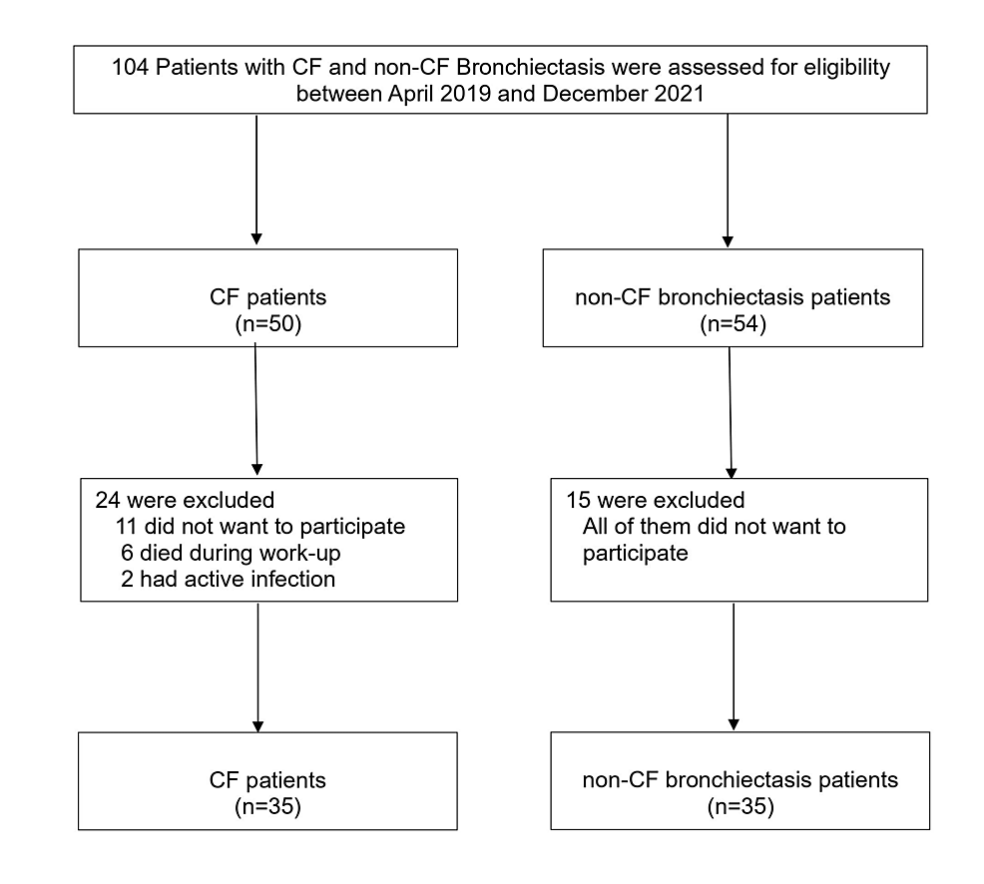
Flow chart of the participants CF: Cystic fibrosis

This prospective cross-sectional study was conducted between April 2019 and December 2021. A total of 104 participants, both CF and non-CF, aged over 18, were monitored at the Adult Chest Diseases Outpatient Clinic of Marmara University Pendik Training and Research Hospital (see Figure 1). From this pool, 34 patients were excluded due to a lack of interest in further investigation, passing away, or having an active respiratory infection during follow-ups. This left a study group of 35 CF and 35 non-CF adults. Data, including the duration since disease diagnosis and socio-demographic information, were recorded. A patient follow-up form was utilized to gather details on their current treatments, comorbid diseases, number of exacerbations, and hospitalizations in the past year. The study also assessed oral nutritional support and oxygen supply usage. Previous year's radiological examinations and sputum sample pyogenic culture results were reviewed. The study was conducted in accordance with the Declaration of Helsinki and approved by the Institutional Ethics Committee of Marmara University (protocol code approval number: 09.2019.503). Informed consent was obtained from all subjects involved in the study.

Pulmonary function testing (PFT)

Each participant underwent PFT using a specific device (JAEGER Masterscreen, Vyaire Medical, Mettawa), calibrated daily by a consistent technician. Recorded metrics included forced expiratory volume in 1 second (FEV1), forced vital capacity (FVC), and forced expiratory flow between 25% and 75% of the FVC (FEF 25-75). PFT results were interpreted as per the European Respiratory Society and American Thoracic Society guidelines [12].

Sleep study

Participants underwent full-night polysomnography (PSG) using the Embletta device (Natus Medical Incorporated, Orlando, USA). The PSG recorded sleep stages, total sleep time, nasal pressure, thoracoabdominal movement, body position, heart rate, oxyhemoglobin saturation (SpO_2_), leg muscle tone, restless leg, and snoring. If the total sleep time was under 240 minutes, a repeat PSG was offered. The study adopted internationally recognized definitions for apnea, hypopnea, and OSA. All sleep studies were assessed according to American Academy of Sleep Medicine's manual of sleep disorders criteria by the same physician, B.B [13].

Epworth Sleepiness Scale (ESS)

The Turkish version of the ESS was utilized to gauge subjective sleepiness [14]. Comprising eight questions, each graded from 0 to 3, the scale totals can range from 0 to 24. A score of 11 or higher indicates excessive daytime sleepiness (EDS).

Charlson score

The Charlson comorbidity index evaluated participants' chronic diseases and associated comorbidities. This index correlates with the mortality rate observed after a 1-year follow-up [15].

Modified Medical Research Council (mMRC) dyspnea scale score

The mMRC dyspnea scale, originally developed by Fletcher et al. in 1940, measured participants' perceived breathlessness [16]. Scores range from 0 to 4, with each point denoting increasing levels of dyspnea.

Statistical analysis

The statistical analysis was conducted using IBM SPSS Statistics for Windows, Version 22 (Released 2013; IBM Corp., Armonk, New York, United States). Data were presented in the following manner: For continuous variables with a normal distribution: mean ± SD (standard deviation), for continuous variables with a non-normal distribution: median (± IQR [interquartile range]) and for categorical variables: numbers (percentages) with a 95% confidence interval (CI). Comparative analyses were undertaken as follows: The Mann-Whitney U test for continuous variables with non-normal distribution, t-test for paired groups' continuous variables with a normal distribution, and a chi-square test (or Fisher's exact test, when appropriate) for categorical variables. Correlation analyses were performed using Pearson correlation for data with a normal distribution and spearman correlation to elucidate the relationships among evaluated parameters. The potential association between sleep apnea and the evaluated parameters was analyzed using logistic regression, with results shown as Odds ratio and a 95% CI. All tests were two-sided, and a p-value of less than 0.05 was deemed statistically significant.

## Results

Clinical characteristics

The clinical characteristics of the participants are detailed in Table 1. The CF group had a marginally higher median age compared to the non-CF group. However, there were no statistically significant differences between the two groups in terms of gender, BMI, and smoking habits. Notably, the CF group had a significantly higher prevalence of diabetes mellitus and pancreatic disease. Additionally, they displayed a longer duration of the disease and experienced more frequent hospitalizations and exacerbations annually (Table 1).

**Table 1:** Clinical and demographic data of the participants (n=70) BMI: Body mass index; CF: cystic fibrosis; IQR: interquartile range; mMRC: Modified Medical Research Council

	CF Bronchiectasis (n=35)	Non-CF Bronchiectasis (n=35)	p-value
Age, years, IQR	27 (22 – 31)	24 (21 – 46)	0.911
Female sex, %	57	54	0.810
BMI kg/m2, IQR	21.1 (19.2 – 23.7)	22.5 (19.6 – 26.4)	0.128
Former or current smoker, %	5.7	20.0	0.074
Comorbidities			
Anemia, %	28.6	20.0	0.403
Diabetes mellitus, %	22.9	5.7	0.040
Pancreatic disease, %	80.0	0.0	0.001
Cardiac disease, %	0.0	5.7	0.151
Osteoporosis, %	8.6	0	0.077
Charlson index	0.0 (0.0-0.0)	0.0 (0.0-1.0)	0.516
Disease duration, years, IQR	22.0 (18.0 – 25.0)	10.0 (10.0 – 21.0)	0.001
Hospitalization per year, IQR	1.0 (0.0-2.0)	0 (0.0-0.0)	0.002
mMRC score, IQR	1.0 (1.0 – 2.0)	1.0 (1.0 – 2.0)	0.366
Exacerbation per year, IQR	2.0 (1.0 – 2.0)	1.0 (0.0 – 2.0)	0.007
Oxygen usage, %	5.7	2.9	0.555

Pulmonary function test results

Table 2 shows the pulmonary capacities of both CF and non-CF participants. Significant differences were noted. The FEV1 L, FEV1 % predicted, FVC% predicted, and FEF 25-75% predicted values were notably lower in CF patients than in non-CF patients. In the CF group, eight patients exhibited no loss in pulmonary capacity. Of the remaining, 14 (40%) had obstructive disease, 24 (69%) had restrictive disease, and 12 (34%) had mixed pulmonary disease. For non-CF BE patients, 11 had no loss in capacity. Of the remaining, 11 exhibited (31.42%) obstructive disease, 18 (51.42%) restrictive disease, and 7 (20%) mixed pulmonary disease.

**Table 2:** Pulmonary function test results of the bronchiectasis patients (n=70) FEV: Forced expiratory volume; FVC: forced vital capacity; CF: cystic fibrosis

	CF Bronchiectasis (n=35)	Non-CF Bronchiectasis (n=35)	p-value
FEV 1, L	1.85 ± 0.86	2.26 ± 0.83	0.052
FEV 1 %, predicted	52.35 ± 22.19	68.26 ± 20.61	0.003
FVC, L	2.65 ± 1.07	3.05 ± 0.91	0.100
FVC %, predicted	63.80 ± 21.96	78.52 ± 15.96	0.003
FEV 1/FVC, %	70.55 ± 12.13	73.45 ± 15.21	0.391
FEF 25 – 75, % predicted	32.80 ± 22.24	49.23 ± 29.68	0.013

Polysomnography results

Table 3 summarizes sleep parameters and values from the sleep study. The median ESS value was lower in CF participants, but the percentage of CF patients with EDS was higher. These differences were not statistically significant (n.s.). No significant variances between the groups were noted for sleep parameters like TST, sleep efficiency, durations, percentages of sleep stages, apnea-hypopnea-index (AHI), and oxygen desaturation index (ODI) levels. However, clear disparities were seen in blood oxygen saturation (SPO_2_) levels and heart rate. Specifically, the CF group had a lower average SPO_2_ and a higher average heart rate compared to the non-CF group.

**Table 3:** Comparison of polysomnography results between CF bronchiectasis and non-CF bronchiectasis patients AHI: Apnea-hypopnea-index; EDS: excessive daytime sleepiness; ESS: Epworth sleepiness scale score; IQR: interquartile range; ODI: oxygen desaturation index; REM: rapid eye movements; TST: total sleep time; WASO: wake after sleep onset; CF: cystic fibrosis

	CF Bronchiectasis (n=35)	Non-CF Bronchiectasis (n=35)	p-value
ESS, IQR	3.0 (1.0 – 7.0)	4.0 (2.0 – 6.0)	0.827
EDS, %	8.6	5.7	0.643
TST, min, IQR	373.8 (330.1 – 396.1)	366.5 (298.5 – 420.3)	0.746
Sleep efficiency, %	83.4 (75.5 – 87.5)	83.5 (73.3 – 91.0)	0.537
Sleep onset, min, IQR	20.3 (12.0 – 45.0)	20.2 (12.6 – 41.7)	0.916
WASO, min, IQR	53.0 (23.5 – 71.5)	40.5 (29.2 – 66.9)	0.257
REM onset, min, IQR	136.5 (85.0 – 203.0)	115.0 (94.5 – 184.0)	0.518
N1, min, IQR	16.0 (9.5 – 25.5)	17.5 (11.5 – 21.5)	1.000
N1, %	4.5 (2.7 – 7.2)	4.8 (3.4 – 6.5)	0.860
N2, min, IQR	167.1 (119.0 – 194.4)	184.0 (137.0 – 235.5)	0.120
N2, %	47.0 (36.7 – 52.4)	52.0 (42.1 – 62.3)	0.034
N3, min, IQR	100.9 (79.0 – 137.0)	99.5 (71.5 – 124.0)	0.431
N3, %	31.0 (24.4 – 37.6)	29.6 (20.4 – 34.9)	0.372
REM, min, IQR	63.0 (37.0 – 90.5)	48.5 (30.0 – 75.0)	0.290
REM, %	16.9 (11.1 – 22.4)	13.3 (9.2 – 20.5)	0.264
Supine AHI, events/h, IQR	8.0 (1.0 – 27.0)	18.0 (2.0 – 44.0)	0.190
REM AHI, events/h, IQR	15.2 (4.7 – 23.6)	11.6 (3.8 – 21.9)	0.605
AHI, events/h, IQR	5.4 (2.4 – 9.7)	5.0 (2.6 – 9.1)	0.796
ODI, events/h, IQR	4.3 (1.1 – 8.4)	2.8 (1.1 – 5.6)	0.304
SPO2, IQR	92.1 (90.1 – 94.4)	94.5 (93.5 – 96.7)	0.001
Lowest SPO2, IQR	87.0 (82.0 – 91.0)	90.0 (87.0 – 92.0)	0.024
Heart rate, IQR	71.4 (65.8 – 77.0)	63.6 (60.6 – 69.6)	0.002

In Table 4, which examines OSA and non-OSA participants, a higher proportion of males were noted among OSA patients compared to non-OSA participants. No significant differences emerged between the groups in terms of demographics, comorbidities, hospitalizations, exacerbations, and nutritional support. However, the duration and percentage of rapid eye movement (REM) sleep were notably higher in the OSA group, while the N2 duration was prolonged in the non-OSA group.

**Table 4:** Comparison of demographic, clinical, and sleep parameters between OSA and non-OSA participants BMI: Body mass index; CF: cystic fibrosis; FEV: forced expiratory volume; FVC: forced vital capacity; MMRC: Modified Medical Research Council; REM: rapid eye movements

	OSA	Non-OSA	p-value
Demographics, clinical characteristics, PFT values			
CF Bronchiectasis, %	54.2	45.8	0.811
Non CF Bronchiectasis, %	51.4	48.6	0.913
Male sex, %	60.0	40.0	0.026
Age, years	30.1 ± 13.1	28.5 ± 10.1	0.576
Duration of disease, years	19.8 ± 8.6	15.5 ± 7.6	0.06
mMMRC	1.4 ± 0.6	1.2 ± 4.3	0.215
BMI, kg/m2	23.1 ±4.4	21.8 ± 3.9	0.184
FEV1 predicted, %	59.4 ± 24.5	61.1 ± 21.0	0.758
FVC predicted, %	69.7 ± 20.3	72.6 ±20.9	0.568
FEV1/FVC	71.4 ± 12.5	72.6 ± 15.1	0.743
Comorbidities			
Anemia, %	24.3	24.2	0.994
Diabetes Mellitus, %	8.1	21.2	0.173
Pancreatic disease, %	37.8	42.4	0.696
Heart disease, %	2.70	3.03	1.00
Osteoporosis, %	2.70	6.06	0.599
Charlson index, %	0.3 ±0.8	0.4 ± 0.7	0.140
Nutritional support, %	18.9	36.4	0.101
O2 support, %	5.40	3.03	1.00
Exacerbation per year	1.5 ±1.2	1.8 ±1.5	0.799
Hospitalization per year	0.6 ± 0.9	0.8 ± 1.4	0.788
Pseudomonas colonization	45.9	57.6	0.543
Sleep parameters			
ESS	4.8 ±3.9	3.7 ± 3.1	0.308
N1, min, IQR	18.6 ±11.3	18.9 ±11.2	0.855
N1, %	5.3 ±3.2	5.6 ±3.2	0.517
N2, min, IQR	165.3 ± 57.6	180.3 ±64.5	0.165
N2, %	44.9±11.5	54.1±18.8	0.010
N3, min, IQR	111.0 ±42.4	93.5 ± 40.5	0.071
N3, %	31.0 ±11.2	30.8 ±15.0	0.510
REM, min	70.2 ±29.8	44.2 ±27.5	0.001
REM, %	18.9 ±6.9	12.2 ±7.2	0.001
Heart rate/min	67.7 ±8.8	69.9 ±9.0	0.309

As detailed in Table 5, both univariate and multivariate regression analyses were conducted. For all patients, male gender and disease duration were identified as significant risk factors for developing sleep apnea. When CF and non-CF BE patients were evaluated separately, significant risk factors diverged between the groups. For CF patients, the univariate model highlighted the mMRC score as significant. For non-CF BE patients, various factors, including male gender, disease duration, FEV1 L, and FVC L, emerged as significant in the univariate analysis. However, in the multivariate analysis, only disease duration remained significant for non-CF participants.

**Table 5:** Multivariate analysis of factors influencing disease types in all participants, cystic fibrosis bronchiectasis, and non-cystic fibrosis bronchiectasis groups FEV: Forced expiratory volume; FVC: forced vital capacity; mMRC: Modified Medical Research Council

All Participants (n=70)			
Variables	Odds Ratio	95 % CI	p-value
Multivariate			
Age, years	1.02	0.98 – 1.07	0.332
Male sex	0.30	0.11 – 0.85	0.023
Disease duration	1.07	1.00 – 1.14	0.041
Cystic Fibrosis Bronchiectasis (n=35)			
Multivariate			
Age, years	1,03	0,86 – 1,23	0,747
Male sex	0,68	0,15 – 2,96	0,603
mMRC score	4,61	0,71 – 30,21	0,109
Non-Cystic Fibrosis Bronchiectasis (n=35)			
Multivariate			
Male sex	0,17	0,01 – 2,21	0,176
Disease duration	1,13	1,00 – 1,28	0,049
FEV1, Lt	3,38	0,37 – 30,71	0,279
FVC, Lt	1,09	0,09 – 13,33	0,947

## Discussion

In our study, we assessed 35 adult patients with CF and another 35 with non-CF BE. We observed that CF patients experienced a higher number of exacerbations, suggesting a greater disease severity within this group. Non-CF patients displayed lower values for FEV1% expected and FVC% expected, and they had endured their condition for a more extended period. Upon evaluating the sleep test results of all participants, we discovered that 37 (or 53%) exhibited symptoms of sleep apnea. Of these, 19 were from the CF group, and 18 were non-CF BE patients. A detailed breakdown revealed that 54% of CF patients and 51% of non-CF BE patients had OSA. This suggests that the specific nature of the BE (whether CF or non-CF) does not particularly predispose patients to sleep apnea.

In a pioneering study using the Pittsburgh Sleep Quality Index on non-CF patients, sleep quality disorders were identified in 56.9% of the 144 cases examined [17]. In another first-of-its-kind study employing PSG, Faria and colleagues discovered OSA in 40.8% of non-CF patients, a figure closely mirroring our findings. In this study, age was not determined to be a risk factor [18]. In contrast, Borekci and his team, through their PSG study, found OSA in 55.8% of 43 non-CF patients, predominantly mild OSA, and demonstrated that the prevalence of OSA escalated with age [19]. However, our results differed. We did not discern a rising trend of OSA with age. Notably, the average age of participants in our study was in the 20s, while Börekçi's research had a median age of 50. The younger age bracket of our participants might explain our contrasting observation regarding the impact of age on OSA.

In the current protocol, we reported the prevalence of OSA as 54%, and in the literature, the prevalence of OSA showed variations from 30% to 80 %. Shakkottai et al. examined 29 children and 23 adult CF patients with PSG, they detected OSA in 79% and found a 3-fold increased OSA risk compared to the control group; this rate was found to be 1.4 times when only the adult group was analyzed [20]. Barbosa et al. performed PSG at home in CF patients under 18 and detected OSA in 32.3% [21]. Spicuzza et al. performed PSG in a hospital for CF patients under 11 and detected OSA in 70% [9]. In a PSG study conducted by Perin et al. with 51 adult CF and 25 healthy patients, AHI was found to be similar in both groups, and only two CF patients (3.9%) met the criteria for OSA [22].

In this study, male gender posed an increased risk for the development of OSA compared to females. Supporting this finding, Faria and colleagues found that male gender is at risk for obstructive upper airway (OUA) in non-CF patients [18]. Furthermore, disease duration is a risk factor for OUA, especially in non-CF patients. In a univariate analysis, Börekçi et al. identified disease duration as a risk factor in this patient group, but this risk disappeared in multivariate analysis [19].

Upon evaluating PFT results, neither FEV1% nor FVC% values indicated significant differences between patients with or without OSA. This observation aligns with Faria et al., who did not find a correlation between the FEV1% value and OUA in adult non-CF patients [18]. Another study, encompassing both pediatric and adult CF patients, similarly reported no association between FEV1% and the AHI [20]. Barbosa et al. noted that FEV1% and FVC% values correlated with periods of sleep where oxygen saturation was below 90% and with average O_2_ saturation values [21]. Echoing this, our data revealed a lower nighttime minimum oxygen saturation in CF patients compared to their non-CF counterparts. In adult non-CF BE patients, both Faria et al. and Millross et al. recorded minimum oxygen saturation of 83.29% and 82.5% respectively [18,23]. Notably, our study identified a correlation between the lowest oxygen saturation levels observed at night and the anticipated FEV1% and FVC% values.

In our research, only 6% (2 out of 35) of non-CF patients were identified with EDS. This contrasts with Faria and colleagues, who reported a notably higher incidence of 53.1%. Intriguingly, while Gao et al. detected a positive ESS test in 31.9% of their 144 non-CF BE patients, they found no significant difference when these results were juxtaposed with healthy participants [17,18]. Börekci and team found a positive ESS test in a striking 74% of their 43 non-CF participants. A possible explanation for these disparities could be the age difference between study cohorts. The average age of non-CF BE patients in the aforementioned studies was considerably older, hovering around 50, compared to the younger demographic in our study, which was in their 20s.

In our study, we observed an average sleep efficiency of 83% for both groups. This aligns closely with Faria and team's findings, which showed a rate of 84% in non-CF patients. However, Börekci and colleagues reported a somewhat diminished efficiency at 71% [18,19]. When examining cystic fibrosis patients, sleep efficiency was pegged at 81% in a study encompassing both pediatric and adult CF participants [19]. Contrarily, Dansey and colleagues, in their PSG assessment of 19 adult CF patients against 10 healthy counterparts, highlighted that the CF group had noticeably poorer sleep efficiency compared to the healthy group (71% versus 93%) [11]. Delving into REM sleep proportions, our results indicate CF patients experienced an average of 16.9% REM sleep relative to total sleep, as opposed to 13.3% in non-CF patients. These figures are in the same ballpark as Faria and team's 17.3% for non-CF BE patients, and the 17.4% noted by Shakkottai and colleagues for CF patients [18,19]. These collective findings underscore the fact that both patient cohorts aren't achieving optimal sleep, underscoring the imperative for proactive screenings.

The limitations of this study include being a single-center study and the absence of upper respiratory diagnoses like sinusitis and polyps in the study variables. The absence of a control group and the small number of patients can be perceived as another negative aspect. Another one is that due to the cross-sectional study structure, the cause-and-effect relationship with the variables cannot be established. The inclusion criterion of no exacerbation in the last month might have excluded more severe lung involvement patients who frequently experience exacerbations, possibly introducing selection bias [5].

## Conclusions

In light of our findings, our study stands out as a comprehensive evaluation of patients' clinical features. As the prevalence of CF patients in adult pulmonology clinics rises, our data reinforce the necessity of promptly conducting sleep studies and swiftly addressing any identified issues. The discovery of OSA in half of our non-CF cohort-mirroring the rates in CF patients highlights the essentiality of meticulously examining sleep patterns in all BE patients, irrespective of the underlying cause. This further underlines the urgency for early sleep test referrals to facilitate timely interventions. Our findings suggest that current treatment protocols for BE patients might need revisiting to optimize patient quality of life. In sum, with the proliferation of sleep laboratories in recent times, further studies in this realm hold the promise of enhancing both the quality and possibly the duration of life for BE patients.
